# Lectures on Nervous Diseases

**Published:** 1889-06

**Authors:** 


**Lectures on Nervous Diseases from the Standpoint of Cerebral and Spinal Localization, and the Later Methods employed in the Diagnosis and Treatment of these Affections.** By AMBROSE L. RANNEY, A. M., M. D., Professor of the Anatomy and Physiology of the Nervous System in the New York Post-Graduate Medical School and Hospital; Professor of Nervous and Mental Diseases in the Medical Department of the University of Vermont; author of the Applied Anatomy of the Nervous System, Practical Medical Anatomy, Electricity in Medicine, etc.; profusely illustrated with original diagrams and sketches in color, by the author; carefully-selected wood cuts, and reproduced photographs of typical cases. Philadelphia: F. A. Davis, publisher. 1888. Royal octavo, cloth; pp. xiv., 728.

This book has been divided into seven sections. Section I. treats of Anatomical, Physiological and Pathological Deductions respecting the Cerebro-Spinal Axis of Man.

In this part of the work we find numerous diagrams, mostly schematic, which serve to convey clearly the author's views. The plates are beautifully executed in colors, and are in accord with the present state of our knowledge.

The only exception to this statement is the plate on page 54, which is meant to illustrate the regions of the cortex of the brain, associated with special parts of the body, as a guide to the seat of destructive processes in connection with motor paralysis or spasm.

The author, in a note, says that in accordance with the views lately advanced by Horsley, this diagram might be modified somewhat. This plate is so incorrect, that it might better have been omitted. The hand center is given much to a greater portion of the ascending parietal convolution.

There is nothing new in the text, which, however, is very clear, and gives in a few pages an excellent idea of the various spinal and cerebral tracts and their respective functions.

Section II. is devoted to the Clinical Examination of Patients Afflicted with Nervous Diseases, and the Steps Employed as Aids in Diagnosis.

This is a most valuable portion of the book, as a large number of medical men have more difficulty with this class of diseases than with any other. Here we find a number of useful hints bearing upon the symptoms revealed by the sense of sight, such as the physiognomy, the appearance of the eye, and the gait and attitude of the patient.

The examination of the eye and the tests of vision are very carefully and thoroughly given. So thorough an advocate of careful examination of the eye is he, that in closing he says: " Personally I have come to regard the examination of any patient sent to me as incomplete until I have tested the state of refraction and accommodation, and examined with care the condition of the ocular muscles. This view has not been hastily formed, and my daily experience has confirmed me in it. I believe the time will come when the tests employed in eye-examination will rank in importance in neurology with the knee-jerk test, which for generations, as Gowers remarks, simply amused school-boys. In this view we can heartily concur. But it seems strange that with all the space given to the eye that no mention is made of the narrowing of the visual field so often observed in hysterical patients. This symptom is a very important one, and should be looked for in all cases of hysteria.

Section III. is devoted to Diseases of the Brain and Its Envelopes.

The subject is tabulated under five heads: 1. Congenital Defects; 2. Diseases of Cerebral Vessels; 3. Inflammatory; 4. Structural Changes; 5. Cerebral Tumors. The description of diseases is good in the main. Multiple sclerosis is, however, slighted. Such prominent symptoms as scanning speech and nystagmus are only hinted at, while the increase of reflexes is not noticed.

In Section IV. we find several good diagrams of the Cord and a number of carefully-selected figures from other authors, illustrating the various deformities which are the result of different cord lesions.

The style is simple and direct. The author, although he describes spinal anemia, clearly does not believe in the existence of this condition. In this we most heartily agree.

In Section V., treating of Functional Nervous Diseases, Professor Ranney shows very decidedly his belief in the theory that these diseases are frequently reflex manifestations. Especially does he pin his faith to the influence of refractive errors in chorea and epilepsy. He also reports a very interesting case of chorea, which was cured by a partial tenotomy of internal rectus. He also reports cases of epilepsy cured by correction of refractive errors, and by partial tenotomies of the muscles of the eye.

In his advocacy of so much surgery of the eye, it seems as if the enthusiasm of the author had carried him a little too far. The principle is, undoubtedly, correct, but we have too many cures on record of patients on whom no tenotomies are performed, to lead us to believe that so large a number of these patients need treatment so heroic. Case III., page 485, is an interesting and valuable record.

In his article on Hysteria, there is an enumeration of surgical operations for the relief of patients, but we are left in doubt as to whether the doctor justifies them or not.

In the last part of the book we find an excellent section on Electricity. Unlike other authors, Prof. Ranney has given considerable space to static electricity, which he advocates for a large number of cases. In his views of this therapeutic agent, he is fully in accord with Charcot and Vigouroux. His discussion of galvanism and faradism, and their therapeutic application, is exhaustive. While many will say that he is too optimistic in his views, nevertheless it is well to have extremists, for there are too many halting skeptics whose influence must be counteracted. The work, as a whole, is a valuable one, and contains much that is not to be found in the writings of others. The publisher's work has been well done, and the attractive appearance of the work gives it an added value.

